# The Vital Roles of Agricultural Crop Residues and Agro-Industrial By-Products to Support Sustainable Livestock Productivity in Subtropical Regions

**DOI:** 10.3390/ani15081184

**Published:** 2025-04-21

**Authors:** Ali Mujtaba Shah, Huiling Zhang, Muhammad Shahid, Huma Ghazal, Ali Raza Shah, Mujahid Niaz, Tehmina Naz, Keshav Ghimire, Naqash Goswami, Wei Shi, Dongxu Xia, Hongxia Zhao

**Affiliations:** 1 Guangdong Provincial Key Laboratory of Animal Nutrition and Regulation, College of Animal Science, South China Agricultural University, Guangzhou 510642, China; alimujtabashah@nwafu.edu.cn; 2 Department of Livestock Production, Shaheed Benazir Butto University of Veterinary and Animal Sciences Sakrand, Sindh 67210, Pakistan; 3 College of Animal Science and Technology, Northwest A&F University, Yangling District, Xianyang 712100, China; huma.ghazal@nwafu.edu.cn (H.G.); naqash@nwafu.edu.cn (N.G.); 4 College of Veterinary Medicine, Inner Mongolia Agricultural University, Hohhot 010018, China; zzhl3425@163.com (H.Z.); hongyuan345@gmail.com (W.S.); xia19861608693@163.com (D.X.); 5 Laboratory of Agricultural and Food Biophysics, Institute of Biophysics, College of Science, Northwest A&F University, Yangling District, Xianyang 712100, China; mshahid@nwafu.edu.cn (M.S.); mujahidniaz@nwafu.edu.cn (M.N.); 6 Khairpur College of Agricultural & Management Science, Sindh Agriculture University Tandojam, Sindh 07005, Pakistan; arshah@sau.edu.pk; 7 Department of Microbiology and Molecular Genetics, Woman University Multan, Multan 59300, Pakistan; tehmina.naz.alpha@gmail.com; 8 College of Economics and Management, Northwest A&F University, No.3 Taicheng Road, Yangling District, Xianyang 712100, China; forestrykeshav@nwafu.edu.cn

**Keywords:** agriculture crop residues, agro-industrial by-products, sustainable livestock production, waste-to-feed, crop residue utilization

## Abstract

Sustainable livestock production is a critical component of global food security and environmental stewardship. Agricultural crop residues, such as cereal straws, stovers, and hulls, as well as agro-industrial by-products, including oilseed meals, distillery wastes, and fruit/vegetable processing residues, are generated in large quantities worldwide, and these residues can be used in the diet of the animals to reduce the feed production cost and sustainability. In this review, we found that the use of treated crop residues in the diet of animals increased the production performance without causing any side effects on their health. Additionally, we also noticed that using these crop residues also mitigates the methane production in ruminants and feed costs, particularly for harvesting the feed crops. Traditionally, these materials have often been underutilized or even disposed of improperly, leading to wastage of valuable nutrients and potential environmental pollution. By incorporating these materials into animal feed formulations, livestock producers can benefit from several key advantages. The review further discusses the challenges and considerations involved in the effective utilization of these alternative feed resources, such as variability in nutrient composition, anti-nutritional factors, and the need for appropriate preprocessing and formulation strategies. Emerging technologies and innovative approaches to optimize the integration of crop residues and by-products into sustainable livestock production systems and also reduce global warming, particularly methane, CO_2_ and other particles that affect the environment after burning these crop residues, are also highlighted. By synthesizing the current knowledge and exploring the multifaceted benefits, this review underscores the vital roles that agricultural crop residues and agro-industrial by-products can play in fostering the sustainability and resilience of livestock production, ultimately contributing to global food security and environmental stewardship.

## Simple Summary

The diet plays a crucial role in the production and health of the animals. The feed of animals contributes a significant cost for farmers. However, the use of agriculture crop residues such as cereal straws, stovers, and hulls, as well as agro-industrial by-products, including oilseed meals, distillery wastes, and fruit/vegetable processing residues can fulfill the requirements of the animals and also reduce the agriculture wastes. In current review we focused on the nutritive value of the different agriculture crop residues and utilization of these crop residues in the diet of the animals to support the sustainable livestock productivity and mitigation of the crop residues waste.

## 1. Introduction

Global population growth has led to an exponential increase in food and energy demand [1]. This escalating demand has necessitated the industrialization and modernization of agro- and food-based industries, thereby enhancing their production diversity and improving the quality and quantity of their products. A concomitant consequence of this development has been the generation of substantial amounts of agro-industrial food waste, which is frequently disposed of in landfills, primarily due to the high costs associated with treatment methods to reduce the organic load from both industrial and domestic sources [2].

Food and Agriculture Organization (FAO) of the United Nations (UN) have explicitly emphasized the role of vegetable waste in contributing to the carbon footprint and fruit waste in creating blue water hotspots (A blue water hotspot refers to a geographic area where freshwater resources (such as rivers, lakes, and aquifers) are under significant stress due to high water demand, scarcity, etc.) [3]. According to reports from the United Nations Environment Programme (UNEP), the economic loss attributed to food waste was estimated to be approximately USD 400 billion (United States Dollars) [4]. These concerns have necessitated the implementation of sustainable and environmentally compatible methods for the optimal utilization of agro-industrial and food waste as a valuable resource to produce bioproducts such as biofuels, organic acids, biopolymers, enzymes, and other commercially significant products, rather than the indiscriminate discharge into the atmosphere that can result in severe environmental pollution [5].

The fodder requirements for animals’ production are crucial, and shortages of fodder cause huge economic losses for farmers. Despite rising demand for animal products, the livestock sector is facing environmental sustainability challenges, supply shortages of feed, and economic viability challenges. Animal products, such as meat, milk, and other animal-derived products, play a crucial role in global food security [6]. In light of rising prices for conventional feed ingredients such as soybean meal and maize, the search for sustainable and cost-effective alternatives has intensified. As a consequence, agricultural crop residues and agro-industrial by-products have emerged as valuable sources of increasing livestock productivity while preserving the environment [7]. A vast amount of crop residues, including straws, stovers, husks, and haulms, are often underutilized or burned, causing pollution. Furthermore, by-products of agricultural processes, such as oilseed cakes, bran, fruit peels, and molasses, can be used as alternative animal feed. The efficient use of these resources can reduce feed costs, minimize waste, and reduce greenhouse gas emissions associated with conventional feed production [7]. Many of these agro-industrial by-products and food wastes possess low nutritional and economic value. Examples of these types of feedstocks include biofuel co-products (distiller grains, sugarcane bagasse, etc.), agro-industrial processing wastes (oil seed meal, soybean hulls, sugar beet pulp, etc.), crop residues (wheat straw, corn stover, etc.), and fruit and vegetable discards, and these are normally used in the diet of the animals [8].

Utilizing food loss and waste in animal diets addresses waste management, food security, resources, and environmental challenges. Livestock as “up-cyclers” play a critical role in the solution to reducing food loss and waste, with the potential to convert inedible foods into high-quality protein in the form of meat, eggs, and milk, while addressing waste management, food security, resources, and environmental challenges [9]. More specifically, the presence of a myriad of microorganisms in the rumen, and to a lesser extent, the large intestine, has the potential to effectively degrade fiber present in human-inedible plants and plant by-products to enable the ruminant host to generate high-quality protein, including essential amino acids and fatty acids [10]. Animal protein is also an important source of B vitamins, with B12 obtained exclusively from animal sources, as well as A, D, and K2 (organ meats) and various minerals (i.e., zinc, selenium, iron) that are often more available in animal than plant-based foods [11]. According to studies, dairy cows fed treated rice straw supplemented with urea-molasses blocks produced comparable milk yields to those on conventional diets, while their feed costs were significantly reduced [12]. Beef cattle are shown to gain more weight and produce a better carcass when fed treated maize stover along with leguminous fodder, demonstrating the potential of these resources when used in intensive production systems [13]. As an alternative to costly protein concentrates, agro-industrial by-products like cottonseed cake, palm kernel meal, and cassava peel have proved to be effective, particularly in smallholder farming systems without access to commercial feeds [14]. Most developing countries burn crop residues, releasing significant amounts of carbon dioxide, methane, and particulate matter. This contributes to climate change and respiratory diseases [15]. As a result of diverting these residues into animal feed, farmers can reduce environmental pollution and simultaneously increase feed availability. Furthermore, by using local by-products, the carbon footprint associated with feed transportation and storage is reduced, resulting in more decentralized and resilient livestock production systems [16]. Globally, agricultural crop residues and agro-industrial by-products can provide an alternative to conventional livestock feed, improving productivity, reducing environmental impacts, and improving farmer livelihoods. With innovative processing technologies, multi-stakeholder collaboration can help unlock the potential of these underutilized resources, contributing to food security and sustainability [16]. In addition to reducing feed costs and improving farm sustainability, agricultural residues can be used as livestock feed. However, these feed residues can be utilized as animal feed and can help farmers reduce their reliance on commercial feed [17]. While residue-based feeding can enhance profitability, challenges like storage losses and competition with alternative uses must be addressed. Cost-effectiveness also depends on factors such as residue quality, processing requirements, and nutritional supplementation [17]. Generally speaking, integrating agricultural residues with livestock systems can enhance farm economics, especially in areas where resources are limited.

The goal of this review is to synthesize current knowledge in this area of agricultural development, highlighting key opportunities, challenges, and research gaps to guide future efforts.

## 2. Agricultural Crops, Agro-Industrial Residues, Types, and Sources

**Figure 1:**
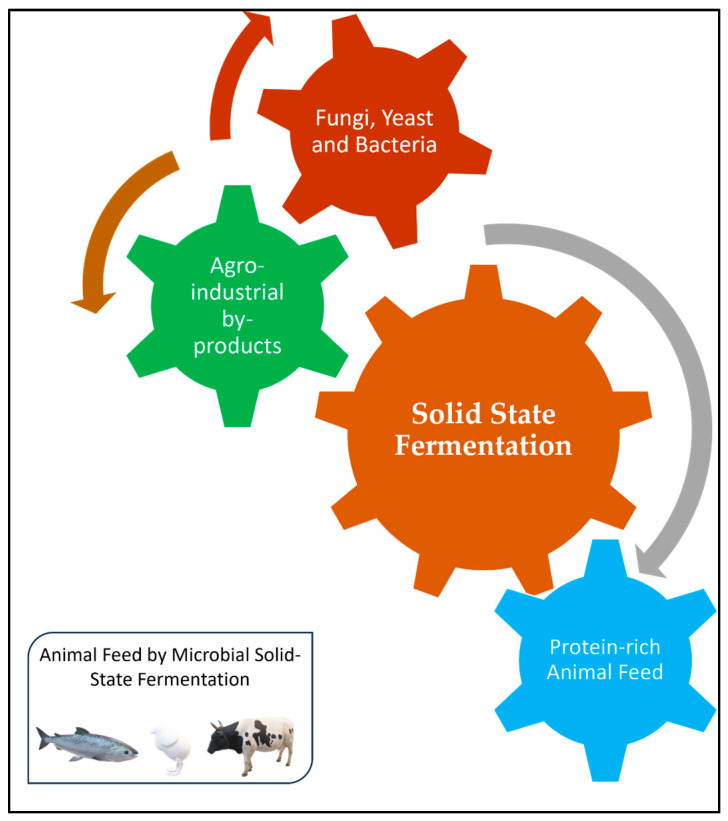
Preparation of feed from agro-industrial waste by using microbes.

Crop residues, also known as primary biomass resources (stalk, leaves, etc.), are inedible plant fragments that are left in the field or orchards after the primary crop has been harvested [18]. Energy crops, seeds, nuts, fruits, veggies, and vegetables are some of the sources from which these leftovers are made. Straw, stover, stubble, stalks, leaves, sticks, haulms, branches, roots, twigs, trims, brushes, and pruning make up the majority of them. Materials left behind after a crop is converted into a primary resource are referred to as agro-industrial residues or secondary biomass resources [19]. Husks, hulls, dust, peels, straws, sawdust, bagasse, pomace, corncobs, and other leftovers from the food and wood processing sectors can be included in the secondary biomass resource. Moreover, tertiary biomass are residues from the raw material processing units [20]. Rice hulls, wheat chaff, corn cobs, and soybean seed coats may be classified as tertiary crop residues. The layout of preparation feed from agro-industrial waste by using microbes is presented in Figure 1.

### 2.1. Rice Straw as Agricultural Residue

The availability of premium forages for ruminant grazing is influenced by the seasons. The animal feed is abundant during the wet season and becomes scarce during the dry season. In countries where feed and high-quality forages are scarce, rice straw remains an affordable, abundant, and practical choice for feeding cattle, buffalo, goats, and sheep [21]. In 2000, FAO estimated that the world produced roughly 2 thousand million tons of cereal straw annually. Furthermore, Southeast Asian nations produce about 200 million tons of straw made from rice each year. For each hectare of barley farm, which symbolizes the total quantity of rice straw output, the weights of grain from rice and rice straw that can be harvested are thought to be equal [22,23].

**Figure 2:**
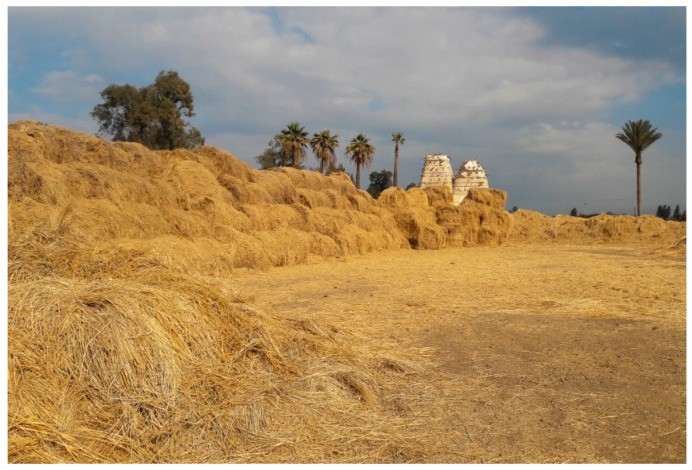
Stock of Rice straw [26].

After harvest seasons, rice straw as well as other agro-industrial wastes are widely available in large numbers in many agricultural regions. Rice farmers produce a lot of rice straw in several agricultural countries. Over 90% of the animal population in Southeast Asia, which includes ruminant populations in Mongolia as well as China, is frequently fed with between 30% and 40% of the rice straw produced in the region [24]. Rice straw typically contains 3–6% protein. It has thick cell walls, biodegradable polysaccharide fractions such as cellulose, hemicellulose, and starch, as well as acid detergent fibers (ADFs) and neutral detergent fibers (NDFs) [25]. It also contains the indigestible phenolic chemical lignin. When fed as fodder, rice straw is primarily used as bulk or filler to assist ruminants in meeting their dry matter requirements and is 80% of energy. Its low crude protein content is approximately 3% to 7%, whereas its high dry matter varies from 92% to 96% [25]. Stock of Rice straw Figure 2.

### 2.2. Corn Stover as Agricultural Residue

**Figure 3:**
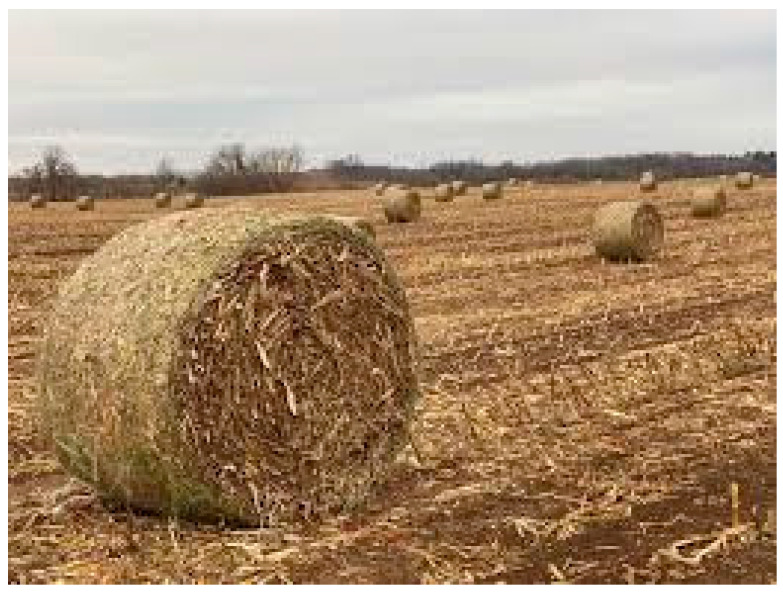
Corn Stover [20].

Corn stover is the branches, leaves, and husks that remain after corn is finished and being harvested. Its principal constituents are cellulose (about 35% *w*/*w*), hemicellulose (approximately 20% *w*/*w*), and lignin (about 12% *w*/*w*). In the United States, tillage is used to incorporate a small quantity of maize stover into the soil to maintain soil productivity [27]. The leftover maize stover is collected and widely used as feed for ruminants and as bedding for cattle. Around half of the total mass of the above-ground part of the plant is stover, and the globe currently harvests more than one billion tons of corn yearly; the harvest index ranges from 47 to 56%. About one billion metric tons of corn stover are produced globally, as one dry kg is produced for every kilogram of dried corn grain [28]. Stock of corn stover Figure 3.

### 2.3. Barley Straw as Agricultural Residue

**Figure 4:**
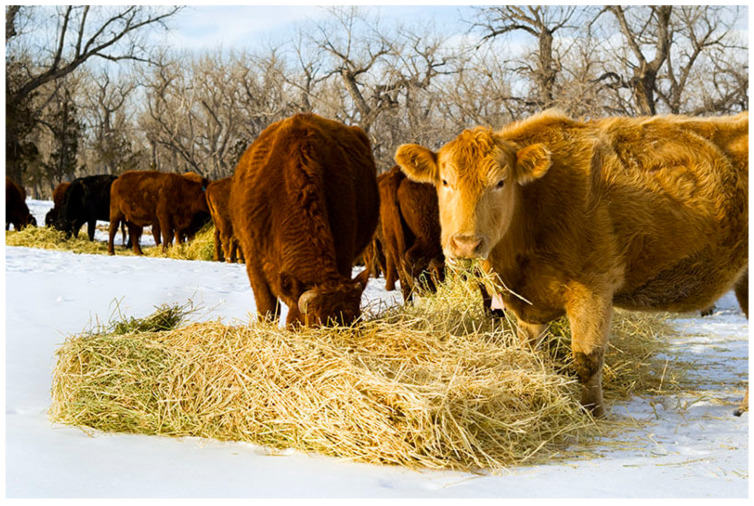
Animals feeding on barley straw [33].

There is growing pressure on the barley-livestock farming system due to increasing incomes, urbanization rates, and animal and human populations [29]. These challenges frequently result in increased land use and continuous cultivation [30]. A decrease in productivity and degradation of the land could result from inadequate agricultural land management [31]. According to reports, soil erosion can be reduced by up to 80% in agricultural field plots when 30% of the wheat is left behind. Barley straw is a valuable resource in the country’s mixed crop-livestock systems. A total of 1.2 metric tons of straw are produced for every metric ton of grain. Barley straw has higher nutrient density than wheat straw, averaging 90.9% dry material, 3.8% protein content as crude protein, and 6 MJ energy available for digestion per kg of dry matter. However, it is high in cellulose and low in calcium and phosphorus. Because ruminal microbes may ferment cell walls, barley straw can be utilized by ruminant animals. Microorganisms found in horse dung are also capable of degrading cellulose [32]. Figure 4 shows animals are feeding on barley straw.

### 2.4. Mixed Crop Leftover as Agricultural Residue

Maize stover, or leftover maize crop, is another tool Ethiopians utilize to feed their cattle and mulch their soil. Extension outreach has been shown to encourage farmers to eliminate more maize combustion on crop plots. Increasing ownership of animals among farmers was linked to increased use of maize stover as feed and fewer people using soil amendment [34]. In Ethiopia, the use of maize stubble is influenced by mixed agricultural systems and planting schedules [34]. Research has also been done on how Ethiopian smallholder farmers use cereal straw and pulses in diversified agricultural systems. The use of breakfast cereal and pulse straws for soil amendment was positively impacted by the farmer’s level of education, the separation that separated the household and the cropping plot, the affordability of agricultural assistance, the farmer’s comprehension of soil modification, the slope of the trimming plot, farmer-to-farmer furtherance, and the stock of crop residue [35].

The feed resources that Ethiopia’s sugar manufacturers have access to, of which sugar top is a major one, are not being completely utilized. Sugarcane tops, an unusual feed, are primarily obtained from privately or state-owned sugarcane farms during cane harvesting season. The leaf, the protective coating, and the newly formed, simple-sugar-rich stem are its three main components [36]. Prior to harvest, the fields’ chosen fire tactics may have partially scorched or wilted the sugarcane top leaf and sheath, which may have an effect on the nutrients it carries and the quantity of biomass it generates. Despite our nation’s sugar businesses growing and creating more products as a result, not much research has been done on the quantity of manufacturing, availability, and feed consumption of sugarcane at bordering sugar facilities [37].

**Figure 5:**
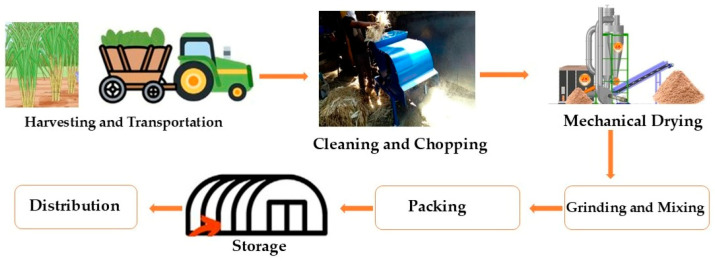
Flow diagram shows processing steps of feed by using Sugarcane Topes.

Sugar cane, one of the major commercial crops grown in tropical regions, especially Asia, is being used as a feedstock to make bioethanol. Due to its large production, bagasse—the fibrous material left over after the juice from sugar cane is extracted—is a significant byproduct that can be used to give ruminants roughage. These days, a large percentage of bagasse is used as fuel to make sugar and jaggery [38]. Additionally, it is a raw material that goes into making boards and paper. Bagasse is not very pleasant and possesses a low nutritional value; hence, its application as animal feed is limited. The principal factors contributing to bagasse’s low nutritional value are several factors, such as low nutrient contents, high polyphenol content, and deficient quantities of minerals, protein, and energy [39]. Much study has been conducted to improve bagasse’s application in a range of manufacturing processes and for the benefit of different livestock species. Because of its weak nutritional content and fibrous nature, bagasse needs to be complemented with other, greater feeds in order to meet animal nutritional needs and even for maintenance. Economic research is necessary since the cost-benefit of employing processed bagasse is toxic, and for feed against non-feed use could serve as a determining factor [40]. The cost of conventional cereal straw, such as wheat, sorghum, or paddy, which is fed to ruminants, would also have an impact on how much bagasse is utilized in animal feeding. Some factors that can seriously damage the usage of bagasse as a nutritional resource include policies that favor biodiesel and provide tax benefits to sugar mills that produce electricity [41]. The composition of the sugarcane and co-products is mentioned in Table 1. The flow diagram shows processing steps of feed by using Sugarcane Topes Figure 5.

Thus, as part of multiple cropping and crop rotation, legumes should occupy a substantial portion of the area now planted to cereals for the purpose of maintaining a robust and sustainable farming system. In many parts of Ethiopia, the soil is deficient in nitrogen, which often lowers crop yields. One of the primary plant components that controls crop productivity is nitrogen, and a deficiency in this element is among the main variables limiting the yield of cereal crops [43]. A number of studies have also pointed out that continuous grain production might result in a decrease in yield, particularly in regions with poor soil fertility, inadequate weed control methods, or a high incidence of soil-borne infections [44].

**Figure 6:**
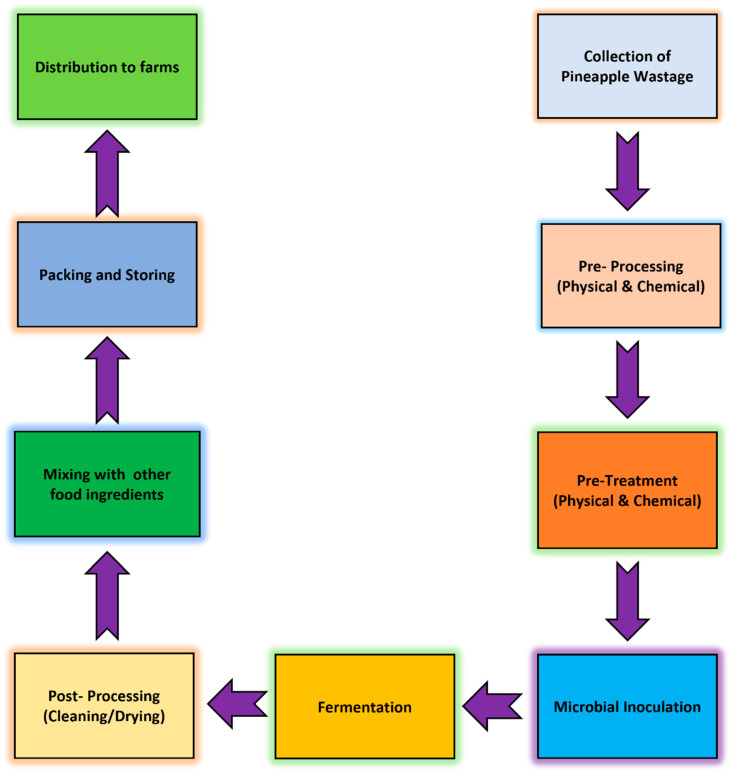
Flowsheet Production of livestock feed by using pineapple wastage.

Ananas comosus, a pineapple species that belongs to the genus Bromeliaceae, has 2000 species. This fruit is an essential source of fiber, vitamins, minerals, carbs, and organic acids for human consumption. Carotenoids, ascorbic acid, and flavonoids are additional antioxidants found in pineapple. Nonetheless, this fruit’s chemical composition varies throughout varieties worldwide [41]. Fresh pineapples can supply up to 17% of your daily need for vitamin C and are rich in the B-complex group, which contains pyridoxine, folate, riboflavin, and niacin. Moreover, bromelain, a stimulant, anti-cancer, and anti-clotting compound, is found in pineapple. A total of 100 g of fresh pineapple contains 86.45 g of water, 48 calories, 12.66 g of carbs, 9.35 g in total sugar, and 1.4 g of fiber [45]. This fruit is also a good source of minerals, such as potassium (151.5 mg), magnesium ions (16.5 mg), the mineral calcium (13 mg), and phosphate (11 mg). Conversely, pineapple has high levels of vitamin C (36.5 mg), riboflavin, thiamine, folic acid, and vitamins A, B6, D, E, and K [46]. Tropical and subtropical climates are the primary locations for the commercial cultivation of pineapple. The leading pineapple-producing countries in the world are China, the countries of Malaysia, Thailand, Kenya, India, the Philippines, Indonesia, and other tropical countries. The literature has acknowledged pineapple waste’s potential as a sustainable substitute feed that could aid farmers in maintaining their crops by reducing feeding costs. Plant cores in peels, farm-grown foliage, and juice-making residues were examples of pineapple by-products [47]. Due to its similar caloric and bioactive chemical composition to lower-quality fruits, the garbage is a perfect substrate for animal feed, particularly for livestock that consumes a diet heavy in fiber and carbs. Many countries, particularly those that produce pineapples, such as Nigeria, Thailand, and India, have reported using the pulp from pineapples as animal feed [45,48]. The functions of the pineapple waste and nutritional values are presented in Table S1 and Table S2, respectively [33,37]. Flowsheet Production of livestock feed by using pineapple wastage (Figure 6) [48].

In Africa and Asia’s southeast, the multipurpose oil palm tree (*Elaeis guineensis jacq*) is commonly planted. While the primary product of oil palm is palm oil, processing byproducts such as palm press dietary fiber, palm kernel icing, and palm oil sludge are also utilized in the animal feed industry [49]. Recently, there has been discussion about using oil palm fronds (OPFs) as a potential feed source for animals that eat herbivores. When mature plants are pruned, they yield 13.3 kg of OPF on average; the yearly production of OPF is roughly 5500 kg/ha [50]. The elements lignin, calcium (Ca), phosphate (P), ether extract (EE), ash, crude protein (CP), crude fiber (CF), and acid detergent fiber (ADF) have all been shown to be present in OPF. OPE has a digestible energy level of 6.5 (MJ/kg DM) and a gross energy of 17.2. These findings suggest that ruminants and other herbivorous organisms livestock could use OPF, a year-round, inexpensive byproduct, as a material source more frequently [51]. The level of inclusion of oil palm frond in animals’ diets is mentioned in Table S3 [52].

Even now, some breweries still malt their own barley to make beer. After kilning, the rootlets that the grains produce during the malting process are separated from the malt. Commonly used as a brewery by-product, spent brewer’s grains are high in protein (over 20) and fiber. They can be fed to animals as supplements, taking the place of more expensive feed ingredients in diet formulations, or in rare cases, they can be eaten by humans. The leftover grain solids from the mashing process are transferred to a holding vessel where they are converted into the brewer’s grain component that is fed to animals. Wort extraction is finished at this point. The next stage of the process is determined by whether the brewer’s grain is sold partially dewatered (BPG), dry (BDG), or wet (BWG) [53].

## 3. Sustainability of Feeding Systems for Ruminants Using Agricultural Biomass

Globally, there is a growing demand for meat and dairy products, for which sustainable livestock production systems are required. Ruminants, such as cattle, sheep, and goats, play an integral role in agricultural systems by providing food, fiber, and other resources [54]. The environmental impact of conventional feeding systems raises concerns about sustainability. Utilizing agricultural biomass as a food resource offers a viable alternative, promoting both economic viability and environmental responsibility [55]. Biomass is a term used for a wide range of organic materials, such as crop residues, forages, and products from food processing. Examples include straw, corn stover, soybean meal, and sugarcane bagasse [55]. Many of these materials, usually considered waste products, can be used as valuable ruminant feed resources. A significant variation exists in the nutritional composition of agricultural biomass, with crop residues typically high in fiber but low in protein. Supplementing these materials with high-protein sources can improve the quality of ruminants’ diets [17]. A legume-based feed can be made more nutritious and tastier by combining it with crop residues. A farmer can reduce feed costs by sourcing feed locally and reducing transportation expenses. By doing this, the farmer supports the local economy as well as boosting farm profitability. Increasing consumer awareness about sustainability has led to the creation of market opportunities for products derived from sustainably fed ruminants. Farmers can earn a premium for adopting such practices by selling meat and dairy products from animals fed on environmentally friendly diets [56].

Although crop residues are widely used to feed livestock, there are still critical gaps in understanding how their optimal use is achieved, especially when it comes to balancing cost and nutrition. There is a disconnect between theoretical potential and practical implementation because of the long-term metabolic and environmental effects on ruminants fed residue-based diets, as well as socioeconomic constraints limiting their adoption among small-scale farmers. In present-day studies, labor inputs, opportunity costs of alternative residue use, and breed-specific responses are often overlooked, resulting in fragmented knowledge that hinders scalable solutions. The development of sustainable residue-based feeding systems requires a holistic, interdisciplinary approach to bridge these gaps, integrating animal nutrition, environmental science, and socioeconomic factors.

A wide range of organic materials from crop production plays a critical role in the feeding systems of ruminants. This approach not only provides for animal nutrition but also promotes sustainable farming. Different types of agricultural biomass are presented in Table 2, and the chemical composition of the different crop residues is mentioned in Table 3.

The term agricultural biomass refers to organic materials derived from agricultural activities, including both primary crops and secondary byproducts.

### 3.1. Types of Crop Residue Used in Feed

#### 3.1.1. Sorghum Residue

In low crop productive zones, crop residues are the dominant feed resources for the livestock. Sorghum is known as the poor man’s crop from the major cereal crops that helps to increase the production of animals in food-insecure areas [59]. In semi-arid and tropical regions, sorghum is one of the important crops. Sorghum stover is highly adoptable in various climatic conditions like high temperature, saline environments, and water scarcity areas, and it has high stress efficiency and proved to be a valuable feed source [60]. Sorghum stover contains cellulose 30.3%, hemicellulose 25.5%, lignin 5.51%, and organic matter 91.5% [60]. Sorghum juice is extracted from fresh sorghum stem pressing, and it is rich in sugar and is highly useful for alcoholic fermentation [61].

#### 3.1.2. Millet Stover

Use of crop residue as feed for ruminants’ production increases in the dry season due to the high cost of conventional feedstuff and improper production of good-quality forages. For feed conservation, ensiling of crop residues helps to increase the fodder quality and nutrition to feed supply over the year [62]. Ensiling of millet stover significantly increases the crude protein. And a study reported that feeding ensiled millet stover to goats in the dry season is the best choice feed for goat production [49,62]. In 2022, ensiled and urea-treated millet stover was used for ram feed in Kenya, and it increased the feed conversion ratio and growth performance of rams. Hence, feeding ensiled and urea-treated millet stover is recommended for growing rams [62].

#### 3.1.3. Sugarcane Bagasse

Sugarcane bagasse is the alternative fibrous and roughage source for ruminants feeding. From one ton of sugarcane, a 300 kg sugarcane bagasse is produced after the juice extraction [63]. Without treatment and processing fresh sugarcane, bagasse was used for animal production, but it has very low nutrient levels. While processing of sugarcane bagasse with mushroom (*Pleurotus florida*) increases the voluntary feed intake, digestibility, and relative palatability. It causes the huge changes in the production of animals [64].

The byproduct remaining after juice is extracted from sugarcane is the sugarcane bagasse. It contains high fiber (40–45% cellulose, 30% hemicellulose, 20% lignin) and protein (3% crude protein). Fermentation of sugarcane bagasse by Lactobacillus and molasses increases the lactic acid production up to 64% and the quality of forages. The feeding of this treated, good-quality bagasse to ruminants increases the animal production [63].

#### 3.1.4. Corn Stover

Corn stover silage and fresh corn stover after corn seed extraction are used for animal feed as crop residue used as feed [65]. Corn stover silage is better in nutrition according to fresh corn stover. Ensiling anaerobic fermentation increases the quality of feed due to the production of lactic acid bacteria and low methane production per unit field of VFAs [66]. Feeding of sorn stover silage did not change the feed intake of goats; it caused the lessening of nutrient utilization. It can be an alternative forage resource to stimulate chewing activity and reduce methane emissions in ruminants [67]. Volatile fatty acids are produced by anaerobic fermentation because carbohydrates are converted into VFA in corn stover [68].

#### 3.1.5. Rice Straw

Rice (*Oryza sativa*) is the staple crop for livelihood in Southeast Asia and more specifically in Vietnam. Meanwhile, ruminant production with approximately 11.2 million heads mainly depends on cut grasses and agricultural by-products since there is a lack of grazing land [69]. Although, in the dry or winter season, cut grasses and pastures only meet about 35–57% of total forage demand, leading to the death of thousands of ruminants, the percentage of rice straw used in ruminant production is really limited compared to its annual yield. In Vietnam, rice straw has not been maximally utilized for ruminant production yet [69]. It is usually fed as part of the forage component in cattle diets during the time when fresh forage is insufficient. For maintaining optimal production levels, feeding only rice straw does not provide enough nutrients to the ruminants [69]. Therefore, increasing the nutritive values of rice straw is very beneficial in the sustainable development of ruminant production. Low and unbalanced nutritive contents, low voluntary intake, and slow rate of digestion are mainly limiting the use of rice straw in ruminant production. For many years, various extensive research studies have attempted to improve the nutritional quality of rice straw as a sustainable source of ruminant forage. The possible alternative for better utilization of rice straw is to improve its nutritive value and digestibility through breaking lignocellulose bonds or at least loosening them to free the major portions of cellulose and hemicellulose to be digested by ruminal microorganisms [70].

Rice straw is one of the major crop residues that is an important source of ruminant’s diet [71]. Rice straw is the common crop straw that contains lignocellulose, polymers, silica, etc. Rice straw has lower nutrient digestibility as compared to corn stover due to the presence of an outer thick cuticle wax layer. Due to this, rice straws were not usually used in animal feed. But urea treatment, co-fermented with probiotics and enzymes, increases the intake and improves the nutrient degradability, VFAs [72]. To improve global feed supply, alternative feeds provide a larger share of animal feed than fresh green forages or roughages. In the case of compound stomach animal production in the dry season, crop residues provide the best alternative that mitigates the methane emission [73,74,75].

#### 3.1.6. Sugarcane Tops

Sugarcane tops are the upper leaves and some parts of sugarcane that are not used in the sugar industry for sugar production. These topes are used for ruminant feed. Anaerobic fermentation of sugarcane tops produces 76.10% neutral detergent fiber and 6.77% crude protein. These are proved as potential roughage sources of livestock feed resources in case of a feed shortage season [76].

#### 3.1.7. Wheat Straw

Wheat straw is considered an abundant lignocellulosic biomass source [77]. Wheat straw is the form of dry roughage having a very low moisture percentage and is the most commonly used of crop residues. Because storage and availability of wheat straw are easy. Use of wheat straw in feed has health benefits like anti-allergic, antioxidant, and anti-inflammatory [78]. A study reported that feeding wheat straw with rumen degradable and undegradable protein improved nutrient digestibility and nitrogen efficiency and prevented high fat accretion in animals bodies [79].

### 3.2. Nutritional Benefits and Economic Implications of Agricultural Biomass in Ruminant Diets

Ruminants require diets high in fiber to maintain optimal rumen function. Agricultural biomass is rich in fibrous materials, which promote rumination and stimulate the production of volatile fatty acids (VFAs), essential for energy metabolism [80]. The fermentation of fiber in the rumen supports the growth of beneficial microorganisms, enhancing digestion and nutrient absorption. While many agricultural residues are low in protein, they can be complemented with high-protein sources to enhance diet quality. For instance, mixing crop residues with legumes can improve the overall protein content, providing essential amino acids necessary for growth and milk production [81]. Certain agricultural biomass sources, such as grain by-products, can be energy-dense. When properly formulated, diets incorporating these energy sources can improve feed efficiency and animal performance [66]. For example, studies have shown that incorporating distiller’s grains in ruminant diets can enhance growth rates and feed conversion ratios [82].

### 3.3. Sustainable Feed Crop Residues Shrink Carbon and Water Footprints

Utilizing agricultural biomass contributes to waste reduction by repurposing materials that would otherwise be discarded [83]. This practice aligns with circular economy principles, promoting resource efficiency and minimizing environmental impact [84]. For example, incorporating crop residues into ruminant diets can reduce the amount of waste generated during harvest, benefiting both the environment and livestock nutrition [85]. Sustainable management of agricultural landscapes, including the integration of biomass in feeding systems, can enhance carbon sequestration in soils. By returning organic matter to the soil, farmers can improve soil health and contribute to climate change mitigation [86]. As mentioned earlier, agricultural crop residues, such as straw, husks, stalks, and leaves, are byproducts of crop production that are often discarded or burned, contributing to environmental pollution. Nevertheless, integrating these residues into animal diets can significantly reduce the environmental impact of livestock farming if they are incorporated into the diet of the animals. By using this practice, not only is waste minimized, but animal production is also reduced, and water resources are conserved [87]. We explore the environmental advantages of feeding crop residues to animals in this section, which focuses on reducing carbon footprints and conserving water. A major contributor to greenhouse gas emissions is livestock production, which accounts for approximately 14.5% of global emissions (FAO, 2013) [88]. Crop residues can mitigate these emissions in several ways, including reduction in carbon footprint (reduction in methane emissions, avoidance of open burning, lower carbon footprint of feed production), water conservation (lower water demand for irrigation for feed crops, water pollution mitigation, increased soil moisture retention) [89].

The digestive process of ruminants, such as cattle and sheep, produces methane during enteric fermentation. In comparison with conventional grain-based feeds, crop residues, especially those rich in lignocellulosic fiber, can alter fermentation patterns in the rumen, reducing methane emissions [90]. Research suggests that crop residues can reduce methane emissions by 10–20% when fed optimally [91].

In order to clear fields, farmers often burn crop residues, releasing CO_2_, black carbon, and other pollutants into the atmosphere. Using these residues for animal feed reduces burning, resulting in a direct reduction in greenhouse gas emissions. The burning of crop residues in India alone contributes to 150 million tons of CO_2_-equivalent emissions each year [92]. Thus, these residues can play a crucial role in mitigating climate change through their use as feed. Traditionally, animal feeds, such as soybean meal and corn, require extensive amounts of land, fertilizer, and energy. Conversely, crop residues are low-input resources, requiring no additional land or fertilizers. By substituting residues for conventional feed, livestock production can reduce its carbon footprint [93].

The feed production of livestock accounts for over 90% of livestock products’ water footprint [94]. It is possible to alleviate water stress in several ways by incorporating crop residues into animal diets. Alfalfa and soy are feed crops that require substantial irrigation. For example, 1 kg of soybean meal requires 2100 L of water [94]. By utilizing crop residues as feed, we reduce freshwater consumption since crop residues are byproducts of already-harvested crops. In feed crop production, excessive fertilizer use leads to nutrient runoff, resulting in the eutrophication of water bodies. Incorporating residues into feed crops minimizes nitrogen and phosphorus pollution [95]. Crop residues improve soil organic matter and water retention. However, partial utilization of crop residues while maintaining some residues for soil health can strike a balance between feed sustainability and soil conservation [96]. Incorporating agricultural crop residues into animal diets is a win-win solution for environmental sustainability. By reducing methane emissions, avoiding residue burning, and reducing the water footprint of livestock production, this practice contributes significantly to reducing climate change and conserving resources. Efforts should be made by policymakers and farmers to promote residue-based feeding systems through incentives, research, and education to maximize these benefits.

## 4. Transforming Crop Residues into High-Value Feed for Improved Meat and Milk Production

Researchers have developed various innovative techniques in recent years to effectively utilize crop residues in ruminant feeding systems. Adding protein-rich feed components, such as oilseed cakes, lentils, or commercial protein supplementation, may significantly improve the nutritional composition of crop residues.

### 4.1. Addition of Supplements in Crop Residues and Blending Crop Residue in the Diet of Cattle/Cow

In a study, researchers investigated the effects of adding soybean meal (SBM) to dietary supplementation on rice straw intake, feed particle size reduction, and food movement through the rumen. The study involved six nonlactating Holstein Friesian cows with ruminal cannula and an average body weight of 660 ± 42.9 kg [97]. The proper protein level is required for the growth performance of cattle. A study reported the use of soya cake in place of fish meal as a protein supplement for growing beef cattle fed urea-treated rice straw did not cause any negative impact on their body growth and performance and maintained the growth performance [98].

Blending crop residue with premium forages such as alfalfa and clover creates a balanced diet. A study finding revealed the lactation efficacy and rumen fermentation properties of dairy cows fed a diet consisting primarily of maize stover in place of alfalfa hay, containing molasses as a supplement. The study recommended the use of an economically beneficial corn stover-based diet supplemented with molasses to feed mid-lactation dairy bovines [99].

### 4.2. Exogenous Fibrolytic Enzymes and Bacteria in the Diet of Cattle/Cows

Common crop residues, such as grass and stalk residues, are hard for ruminants to digest because they contain stiff fibers and complex sugars. Exogenous fibrolytic enzymes have been the subject of a lot of research to find ways to make feed easier for animals to digest [100]. A study found that administering a fibrolytic enzyme product to alfalfa hay during baling reduced the hay’s aerobic stability while increasing NDF (neutral detergent fiber) and hemicellulose contents, as well as total tract digestibility [101]. Feng et al. (1996) reported that adding enzyme supplements to diets while feeding increased cattle’s average daily gain (ADG) by improving consumption and digestibility [102]. Beauchemin et al. (2003) suggested four primary elements contributing to the multifactorial inconsistencies observed in animal reactions to additional enzymes: the enzyme’s features, the forage, the animal, and the management [100].

Treating crop residues such as maize stover, wheat straw, and rice straw with enzymes and bacteria improves their suitability for feeding ruminants. For a long time, enzymes have been a beneficial way to improve the efficiency of pig and chicken feed. In recent years, corn plant silage has become a popular choice to feed ruminants due to its excellent ensiling properties, energy level, and biomass yield [103]. A study found that blending corn silage with complicated lactic acid bacteria quantitatively increased the average daily gain of growing-finishing bulls. This resulted in considerable improvements in daily dry matter consumption, ruminal ammonia nitrogen, and blood urea nitrogen [82]. Researchers have documented that fibrolytic enzyme cocktails enhance the anaerobic use and biodegradation of fibrous crop residues. Researchers treated fibrous feeds with 3 mL/kg DM enzyme level, enhancing the biodegradation of DM, NDF, and ADF above 1 mL/kg DM enzyme level [104]. Ruminants use cellulases and other enzymes for food digestion [76]. When cellulases, xylanases, and ferulic acid esterase (FAE) are mixed, they can break down the cross-links in crop residues very well. Applying these additives before bundling the forage may allow them ample time to interact with the cell wall [105,106].

When external amylase is added to whole-crop maize silages, it aids in the fermentation of both non-fermentable carbohydrates (NFCs) and fermentable carbohydrates (FCs) at all stages of plant development [107]. Enzymes have been utilized for many years to enhance the utilization of poultry and swine feeds. Adding fibrolytic enzymes to silage should boost its homolactic fermentation, decrease proteolysis and dry matter (DM) losses, and make the feed more digestible.

Sugarcane is a semi-perennial tropical grass. Proper harvesting and post-harvest management can extend its productive lifespan [108]. It is commonly utilized to feed dairy and beef cattle and corresponds with the scarcity of pasture [109]. Nevertheless, this fodder might be ensiled to save on the daily labor-intensive tasks of harvesting, chopping, and transporting, as well as crop loss due to mistaken fire. To investigate their impact on the nutritional content of silage, the fibrolytic enzymes β-glucanase and xylanase, along with *Lactobacillus buchneri* 40788, were treated with chopped sugarcane. Elevated acetic acid and decreased ethanol concentrations were observed in treated silages. NDF concentration was considerably lower in total mixed rations containing inoculated silages, and as a result, IVDMD (in vitro dairy microbiological digestibility) was higher. It was concluded that incubating *L. buchneri* into sugarcane ensilage can change the fermentation pattern by increasing acetic acid output, lowering silage nutrient losses, and enhancing bull feed efficiency [110].

### 4.3. Combination of Different Feed Residues and Use of Micronutrients in the Diet of the Animals

Agro-industrial byproducts can be economically and environmentally beneficially incorporated into animal diets for livestock production, particularly for ruminants that might benefit from low-quality, high-fiber feedstuffs. An investigation was conducted using the rumen-simulation technique to study the effects of incorporating grape seed meal (GSM) into dried distiller’s grains plus soluble (DDGS) as protein and energy sources in the diet. The findings suggested that DDGS supplemented with GSM positively regulates ruminal fermentation by reducing the production of methane while having no negative impact on the breakdown of fiber [111]. Reports indicated a depletion of Cu, Zn, P, and S in soil, plants, and dairy cows, with most crop residues lacking Cu and Zn [112,113]. A study was conducted on twelve adult *Jalauni* sheep to investigate the impact of feeding micro-nutrient-rich sorghum hay on nutrient intake and utilization. Supplementing additional minerals with sorghum hay, rich in micronutrients, did not significantly alter the consumption of dry matter. Adding micronutrients to fodder sorghum (Cu, Zn, and Mn) made adult sheep retain more minerals (Cu 2.84 vs. 3.72 mg/d, Zn 11.72 vs. 15.46 mg/d, and Mn 26.19 vs. 40.28 mg/d) without changing the absorption coefficient much [114].

The study was conducted to check the effects of feeding sheep rice straw together with an extra block lick of urea molasses on their performance. Six indigenous sheep, roughly two years old and with an average weight of 12.88 kg, were selected. The survey found that the average daily increase in the live weight of sheep was between 41 and 70 g. The study revealed that the block lick can make better use of crop residues when fed rice straw mixed with urea molasses [115]. Buffaloes that receive inadequate nutritional supplementation often exhibit reduced milk output, poor behavior, poor seasonal breeding, extended calving intervals, anestrus, and low growth rates [116,117]. Researchers conducted a study to assess the impact of ultra-molasses mineral block (UMMB) on 60 buffaloes in field settings. On average, a buffalo consumed 375 g of mineral blocks, including urea molasses, on a daily average. When compared to the pretreatment level, the total milk output increased by 1.02 L (13.21%) per day. As a result, providing nursing buffaloes with UMMB licks increased their milk production and buffalo farming income [118]. Rice straw It is a common agricultural waste in most tropical and subtropical countries. Despite its low crude protein (CP) content and poor rumen fermentation, ruminants feed it [24]. An isotope dilution method with (U-^13^C) glucose and (1-^13^C) leucine was performed on sheep to assess the impacts of rice straw fortified with urea and molasses (R SUM-diet) on plasma glucose and Leu turnover rates. The control group sheep were fed with mixed hay (MH-diet). It was found that the kinetics of plasma glucose and Leu metabolism were consistent between the RSUM diet and the MH diet, and rumen fermentation properties were improved in sheep fed the RSUM diet [119].

Rice straw is the most common agricultural waste in tropical countries, particularly in Asia, and farmers frequently store it for use as ruminant feed. However, the nutritional value of rice straw is negligible. Rice straw has a low protein level (2–5% dry matter), high fiber and lignin content (neutral detergent fiber-nitrogen 50%), poor voluntary feed intake (1.5–2.0%), and low DM digestibility (65%) [120]. Three treatments of rice straw were given to multiparous Holstein crossbred dairy cows: T1 equaled untreated rice straw; T2 was 5.5% untreated rice straw (5 g urea in 100 mL water to 100 g air-dry (91% dry matter) straw); T3 was 2.2% urea plus 2.2% calcium hydroxide treated rice straw (2.0 g urea and 2.0 g Ca(OH)_2_ in 100 mL to 100 g air-dry (91% dry matter) straw). The results showed that applying 2.2% urea + 2.2% calcium hydroxide-treated rice straw and 5.5% urea-treated rice straw significantly increased dry matter intake and digestibility. The results showed that applying 2.2% urea + 2.2% calcium hydroxide-treated rice straw and 5.5% urea-treated rice straw significantly increased dry matter intake and digestibility [121]. Urea-ammonia (5% *w*/*w*) treatment of rice straw led to a 46% increase in digestible organic matter intake compared to untreated straw. This resulted in a 17% improvement in OM digestibility and a 25% increase in voluntary consumption. The treated diet resulted in a 24% increase in the rumen’s pool of volatile fatty acids [122]. Treated rice straw significantly improves digestibility compared to untreated straw by breaking down lignin and increasing fermentable fiber, enhancing rumen microbial activity and nutrient absorption. Chemical treatment also boosts crude protein content, further supporting better feed utilization in dairy cows [24].

Wheat straw is a low-density fodder with a low protein and calorie content. Lactation research was carried out on cows to investigate the effects of total mixed meals based on wheat straw supplemented with *Prosopis juliflora* pods. Total mixed rations increased milk yield by 26% compared to the control group. The effects of dietary interventions on milk constituents such as total solids in milk, solids-not-fat, protein, phosphorus, and calcium were not observed. It was found that giving a total mixed ration to crossbred cows, as opposed to concentrates and roughage (wheat straw), independently boosted feed intake and milk production [123].

### 4.4. Effective Utilization of Agricultural Crop Residues in Enhancing Meat and Milk Production

Utilizing agricultural crop residues for animal production is increasingly vital for sustainable agriculture. Incorporating these residues into livestock feed can reduce dependence on purchased/conventional feeds, thus lowering production costs [124]. This approach significantly enhances livestock productivity, particularly in meat and milk production. Cereal crop residues, such as straw, stover, and husks, are valuable feed resources for ruminants, especially in regions where green forage is scarce at certain times of the year. After harvesting, wheat and rice straws and maize stover become readily available for animal feeding. Rice straw, in particular, is extensively used as ruminant feed [26]. Although biological treatments have improved the nutritional value of crop residues, many remain uneconomical, and processes are not yet fully optimized for field conditions [124,125]. This practice not only reduces reliance on conventional feed but also addresses waste management issues by recycling agricultural by-products. Incorporating crop residues into livestock diets improves nutrient utilization efficiency and promotes a circular economy in agriculture, leading to more sustainable and resilient farming systems.

Crop residues alone are insufficient to meet the nutritional needs of high-yielding animals, such as milking cows, but their value can be enhanced with appropriate supplements [126]. Incorporating crop residues into livestock diets also reduces dependence on conventional feeds, which are often costly and subject to market fluctuations. The production of fibrous crop residues is influenced by various factors, including alternative energy sources, climate, soil and water quality, land topography, animal production systems, technological advancements, and political and economic constraints.

Globally, crop residues represent a substantial portion of agricultural biomass. The global production of residues from six major crops: barley, maize, rice, soybean, sugarcane, and wheat, across 227 countries is estimated at 3.7 petagrams of dry matter per year. Regions including North and South America, Eastern, Southeastern, and Southern Asia, and Eastern Europe each produce over 200 teragrams annually [127]. China is the largest producer, generating over 897.06 metric tons of agricultural crop residues [128]. These residues, including straw, stalks, husks, and other plant materials, are primarily used for animal feed, bioenergy production, soil improvement, and as raw materials in various industries. India follows as the second-largest producer, with over 130 million tons of paddy straw, about half of which is used as fodder [129]. Iran, due to its climatic diversity and abundant natural resources, is among the top producers of over 20 agricultural products, with an estimated 24.3 million tons of agricultural residues annually [130]. On average, producing 1 kg of boneless meat requires 2.8 kg of human-edible feed in ruminant systems and 3.2 kg in monogastric systems [131].

Crop residues are typically low in protein and high in fiber, which limits their digestibility compared to other feed resources. However, their nutritional value can be significantly improved through various treatments and supplementation strategies [132]. Chemical treatments, such as alkalis, ammonia, or urea application, can break down the fibrous content, enhancing digestibility. Biological methods, including the use of enzymes or microbial inoculants, can further improve nutrient availability. Physical treatments, such as grinding or pelleting, also increase the surface area for microbial action, making the residues more palatable and easier for animals to digest. By combining these methods, crop residues can be transformed into more efficient and valuable feed resources, contributing to sustainable livestock production. This multi-faceted approach not only improves animal nutrition but also supports a more sustainable agricultural system by maximizing the use of available biomass. Below, we briefly discuss the various methods widely adopted to enhance the digestibility and nutrient content of crop residues [132].

Chemical Treatments: Chemical treatments of crop residues involve the application of chemicals to improve their nutritional value by enhancing digestibility and nutrient availability [133]. These treatments are particularly important for livestock feed, as untreated residues like straw and stover are often low in protein and high in indigestible fiber, making them less suitable for high-yielding animals. The essential purpose of this method is to break down lignin, which brings up benefits like increased digestibility and enhanced nutrient absorption. It is noteworthy to mention that lignin is a complex polymer found in plant cell walls that provides structural support but makes the plant material difficult to digest. Chemical treatments, particularly with alkalis, break down lignin, exposing the cellulose and hemicellulose fibers that are more easily digested by ruminants [134]. By breaking down the lignin, the cellulose becomes more accessible to the digestive enzymes in the animal’s stomach, particularly in ruminants, where microbial fermentation plays a key role in digestion. This results in improved feed efficiency and higher energy intake from the same amount of crop residue. Chemical treatments can also enhance the availability of other nutrients trapped within the plant cell walls, leading to better overall nutrient absorption and utilization by the animals. The importance of the use of the agricultural residues in the diet of animals is summarized in Table 4.

There are basically 3 widely practiced chemical treatment methods, viz., alkali treatments, oxidative treatments, and acidic treatments. Treating with sodium hydroxide, ammonia, and calcium hydroxide agents falls down in the alkali treatment approach. Sodium Hydroxide (NaOH) is one of the most common chemicals used to treat crop residues. It reacts with lignin, breaking it down into simpler compounds that are more easily degraded by microbial activity in the rumen [112]. Consequently, the treated residue becomes softer, more digestible, and has higher energy content. Meanwhile, ammonia (NH_3_) treatment is another widely used method, especially for its dual benefit of increasing nitrogen content (which enhances protein levels) and breaking down lignin [135,136]. This method is often applied as anhydrous ammonia or urea, which converts to ammonia in the presence of moisture. Ammonia-treated straw or stover shows significantly improved digestibility and protein content. In addition to the above-mentioned two alkali agents, calcium hydroxide, also known as slaked lime, has been used as well, however, it is less common than sodium hydroxide. It functions similarly in breaking down lignin but is generally considered less effective.

In the oxidative treatment method, hydrogen peroxide (H_2_O_2_) and ozone (O_3_) are widely used [137]. This approach uses the oxidative power of hydrogen peroxide to break down lignin. While being practical, it is less commonly used due to the higher cost and handling complexities associated with hydrogen peroxide. Meanwhile, ozone treatment is another oxidative method that can break down lignin. However, it is more experimental and not widely adopted due to cost and the need for specialized equipment. Acid treatment is less common due to the corrosive nature of acids and the potential risks involved. However, it can be effective in breaking down lignin and hemicellulose. Dilute acid treatments are sometimes used in combination with other methods to increase efficacy.

Biological treatments utilize microorganisms, such as fungi, bacteria, or enzymes, to break down the complex lignin structure in crop residues. This process, known as biodegradation, enhances the digestibility of the residues, making them more suitable as livestock feed. Biological treatments are considered more environmentally friendly compared to chemical treatments and can also increase the protein content of the treated material.

In fungi treatment, white-rot fungi are particularly effective at degrading lignin due to their production of ligninolytic enzymes such as laccases, peroxidases, and manganese peroxidases. Species like Pleurotus (oyster mushrooms) [138] and Phanerochaete chrysosporium [139] are commonly used in the biodegradation of crop residues. The fungi break down lignin and hemicellulose while leaving cellulose relatively intact, which improves the fiber’s digestibility. Solid-state fermentation is another widely practiced approach in which crop residues are inoculated with fungi in a controlled environment where moisture content, temperature, and aeration are carefully regulated. Over time, the fungi colonize the residues, breaking down the lignin and increasing the material’s digestibility and nutritional value.

Bacterial treatments include treatment with rumen microbes and other enzymatic additives. Rumen microbes, which naturally occur in the digestive systems of ruminants, can be cultured and applied to crop residues to initiate the breakdown of fibrous materials. These microbes produce cellulolytic enzymes that degrade cellulose and hemicellulose, enhancing the feed’s nutritional profile. On the other hand, commercially prepared enzymes, such as cellulases and xylanases, can be added to crop residues to facilitate the breakdown of complex carbohydrates. These enzymes help to reduce fiber content and increase the availability of simple sugars, which are more easily digested by livestock.

The biodegradation mechanism provides multiple benefits as well. For instance, lignin is a highly recalcitrant polymer that protects cellulose and hemicellulose from microbial attack. The enzymes produced by fungi and bacteria degrade lignin, which allows the more digestible cellulose and hemicellulose to become accessible. This process not only improves the digestibility of the crop residues but also reduces their fiber content, making them more palatable to livestock. Concurrently, some fungi, such as Trichoderma and Aspergillus species, can also increase the protein content of crop residues by converting the fibrous material into microbial biomass [140]. This microbial biomass is rich in protein, adding nutritional value to the treated residues as well.

Physical treatments are mechanical processes used to alter the physical structure of crop residues, making them more digestible for livestock. These treatments include chopping, grinding, pelleting, and other methods that reduce the particle size of the residues, increasing their surface area and making them easier for animals to consume and digest. While effective in improving digestibility, physical treatments can be energy-intensive and costly, particularly for large-scale operations [132].

Chopping is one of the key physical treatment methods. It involves cutting crop residues into smaller pieces using mechanical equipment such as choppers or forage harvesters. This process reduces the length of fibrous materials, making them easier for animals to chew and mix with saliva, which aids in digestion. Chopping improves the intake and digestibility of crop residues by reducing the time required for ruminants to break down the fibers [141]. It also helps in better mixing with other feed components when preparing a balanced diet. Meanwhile, grinding involves breaking down crop residues into fine particles using mills or grinders. The finer the grind, the larger the surface area exposed to digestive enzymes in the animal’s stomach [141].

Grinding significantly improves the digestibility of crop residues by reducing particle size, which enhances the efficiency of microbial digestion in the rumen [142]. It also helps in creating uniform feed mixtures, improving nutrient distribution, and feed intake. Pelleting, on the other hand, compresses ground or chopped crop residues into dense, uniform pellets using a pellet mill. The process involves applying heat and pressure, which can also partially gelatinize starches, improving the overall digestibility of the feed. It enhances the ease of handling, storage, and transportation of crop residues. Pelleted feed is also more palatable to livestock and reduces feed wastage. The process increases the density of the feed, allowing for more efficient use of storage space [142].

India, being a predominantly agricultural country, generates a substantial amount of crop residues every year. These residues, often considered waste products, are a valuable resource for livestock feed, particularly for ruminants like cattle, buffalo, sheep, and goats. On average, India annually generates 500 Mt [143] of crop residue, the majority of which is used as fodder. Crop residues in India mainly include wheat, paddy straw, maize, millets, sugarcane, fiber crops like jute & cotton, pulses, and oilseed farming. Out of various crops grown, rice, wheat, and sugarcane are prone to fodder use. Crop residues such as paddy straw and wheat straw are commonly used as fodder for livestock, particularly in regions where green fodder is scarce. The use of crop residues has been shown to improve the milk yield of dairy cows, particularly when supplemented with protein-rich feeds. In addition, the use of crop residues helps to reduce feed costs, which is particularly important for small-scale farmers who may not have access to expensive commercial feeds. Sugarcane residue (mainly leaves) in India comprises 2% of crop residue, which is around 133 million tons (2017–2018). It is a rich source of carbohydrates, which are essential for energy production in livestock. However, it is important to note that sugarcane residue is low in protein, so it needs to be supplemented with protein-rich feeds to provide a balanced diet for animals. Among different livestock species, ruminants (like cattle and sheep) are generally better equipped to digest fibrous materials like sugarcane residue [143].

*India*: is the largest producer of areca nuts. In regions where areca nuts are cultivated, the outer coverings (sheaths) are often discarded due to their slow decomposition. Scholars investigated the possibility of using these sheaths as livestock feed. They dried, shredded, and combined the sheaths with other feed ingredients to create a complete feed mixture. The study revealed that areca sheaths could entirely replace traditional rice straws in cattle feed. This substitution not only provided better nutrition but also increased milk production and quality [144].

*China*: China has also made significant progress in utilizing crop residues for livestock production. In the northeastern region of China, corn stover is commonly used as a feed resource for beef cattle. The use of corn stover has been shown to improve the growth rates of cattle, leading to increased meat production. In addition, the use of crop residues helps to reduce the environmental impact of livestock production by reducing the need for synthetic fertilizers and reducing greenhouse gas emissions [145].

*Africa*: In sub-Saharan Africa, the use of crop residues as livestock feed is becoming increasingly common, particularly in regions where traditional grazing lands are being converted to crop production [146]. In Ethiopia, for example, the use of crop residues such as teff straw and maize stover has been shown to improve the productivity of smallholder dairy farms. The use of crop residues helps to bridge the gap between the dry and rainy seasons, ensuring that livestock have access to adequate nutrition year-round [147].

**Table 4:** Use of different feed residues in the diet of different animals and their impact on production performance.

S.No	Species	Diet + Dose	Duration	Findings	References
01	Sheep (lamb)	Cabbage leaves 100 g/kg + basal diet	60 days	Reduced the DMI, FCR, growth performance	[148]
02	Goats	Potato leaves (vine) + basal diet/poor quality hay	70 days	Increased feed intake, FCR, digestibility, carcass weight	[149]
03	Sheep	sorghum stovers (Phule chitra cultivar)	120 days	Increased Feed intake, Digestibility, body weight	[150]
04	Fish (Nile tilapia)	Sugarcane bagasse (SB) (20 g kg^−1^)	--	Increased growth performance, immunity, and antioxidant profile	[151]
05	Poultry	Sugarcane bagasse (Lignin)	--	Improved GIT bacterial growth, Health	[152]
06	Sheep	Sugarcane bagasse/rice husk treated with *Trichoderma viride*	--	Increased feed intake apparent digestibility, improved growth performance	[153]
07	Dairy cows	Sugarcane bagasse *Lactobacillus casei* TH14, cellulase, and molasses	--	Increased DMI, fibre digestibility, blood glucose, gross and metabolized energy	[154]
08	Pigs	Sugarcane bagasse treated lignocellulose 30 g/kg	42 days	Improved the gut health and production of butyrate formation, reduced the pathogenic bacteria	[155]
09	Sheep	Corn Stover 100 g/kg	56 days	No any negative effect on growth performance, DMI, digestibility	[156]
10	Dairy cows	Corn Stover + corn gluten (3.4%, 6.9%)	14 days	Increased milk production and quality, lactation performance	[157]

## 5. Anti-Nutritional Factors and Their Mitigation

Plants and other food sources naturally produce chemicals through various metabolic processes, known as antinutritional factors. When ingested, these compounds reduce the body’s ability to absorb nutrients or interfere with the efficient use of specific nutrients, including vitamins, proteins, and minerals. It is crucial to understand that these factors are not intrinsic to the food itself but are influenced by the digestive system of the consuming animal [158].

Certain agro-industrial byproducts, such as soybean meal (SBM), canola or rapeseed meal, and cottonseed meal, contain high concentrations of anti-nutritional factors. In SBM, the main anti-nutritional compounds are trypsin inhibitors, which hinder the function of trypsin, a protein-degrading enzyme, resulting in reduced dietary protein digestibility [159]. SBM also contains allergenic proteins like β-conglycinin and glycinin, which can cause allergic responses, including hypersensitivity and digestive issues in young pigs upon initial exposure to dietary soy protein, limiting SBM use in early-weaned pig diets [160]. SBM also contains oligosaccharides such as raffinose and stachyose, which monogastric animals cannot digest with endogenous enzymes. However, gut bacteria can utilize these to some extent without adversely affecting nutrient digestibility in young pigs [161] and may alter microbiota structure and decrease the production of undesirable compounds in broilers [162].

Glucosinolates (GLS) are the primary anti-nutritional factors in canola or rapeseed meal. These sulfur-containing secondary metabolites are commonly found in plants of the Brassicaceae family, including rapeseed, mustard, and cabbage [100]. While intact GLS are harmless to animals, their breakdown products (thiocyanate, isothiocyanate, oxazolidinethione, and nitriles), produced by the myrosinase enzyme or physiochemical factors like heat and pH, can impair feed intake, growth performance, and liver and kidney function [163]. Sinapine, another anti-nutritional factor in canola or rapeseed meal, is a bitter-tasting phenolic compound that may reduce animal feed consumption [164].

### 5.1. Challenges in the Utilization of Crop Residues

Despite the potential benefits, there are several challenges associated with the use of agricultural crop residues in meat and milk production. These challenges include variability in the availability and quality of crop residues, the need for appropriate treatment and supplementation, and the economic viability of using crop residues as feed resources [165].

#### 5.1.1. Availability and Quality

The availability and quality of crop residues can vary significantly depending on the region, crop type, and farming practices. For example, residues from cereal crops such as wheat and rice are more commonly used as feed resources compared to residues from other crops such as legumes. In addition, the quality of crop residues can be affected by factors such as harvesting methods, storage conditions, and weather patterns [166].

#### 5.1.2. Treatment and Supplementation

As previously mentioned, crop residues are generally low in protein and high in fiber, which can limit their nutritional value. To maximize their potential as feed resources, crop residues often need to be treated or supplemented with other feed resources. However, the cost and availability of these treatments and supplements can be a barrier for small-scale farmers, particularly in developing regions [167].

#### 5.1.3. Economic Viability

The economic viability of using crop residues as feed resources depends on several factors, including the cost of treatment and supplementation, the availability of alternative feed resources, and the market prices for meat and milk. In some cases, the cost of treating and supplementing crop residues may outweigh the benefits, particularly if alternative feed resources are available at lower costs [167].

#### 5.1.4. Environmental and Economic Benefits

The use of crop residues in livestock production offers several environmental and economic benefits. By recycling agricultural by-products, the use of crop residues helps to reduce waste and promotes a circular economy in agriculture. In addition, the use of crop residues can help to reduce the environmental impact of livestock production by reducing the need for synthetic fertilizers and reducing greenhouse gas emissions [167].

#### 5.1.5. Waste Reduction

The use of crop residues as feed resources helps to reduce the amount of agricultural waste that would otherwise need to be disposed of. This is particularly important in regions where waste management infrastructure is limited. By recycling crop residues, farmers can reduce their reliance on landfills and incineration, which can have negative environmental impacts [168].

Circular Economy: The integration of crop residues into livestock diets promotes a circular economy in agriculture by closing the loop between crop production and livestock production. By recycling crop residues, farmers can reduce their reliance on external inputs such as synthetic fertilizers and commercial feeds. This not only reduces costs but also promotes more sustainable and resilient farming systems [168].

#### 5.1.6. Environmental Impact

The use of crop residues in livestock production can help to reduce the environmental impact of livestock production by reducing the need for synthetic fertilizers and reducing greenhouse gas emissions. For example, the use of crop residues can help to reduce the need for nitrogen fertilizers, which are a major source of greenhouse gas emissions in agriculture. In addition, the use of crop residues can help to reduce methane emissions from livestock by improving the digestibility of the feed [169].

#### 5.1.7. Technological Innovations and Future Prospects

The future of crop residue utilization in livestock production looks promising, with ongoing research and technological advancements offering new opportunities to optimize this practice. Innovations in treatment methods, feed formulations, and livestock management systems are expected to enhance the efficiency and sustainability of using crop residues as feed resources [170].

Advanced Treatment Technologies: Emerging technologies, such as enzyme treatments and microbial inoculants, are being developed to improve the digestibility and nutrient content of crop residues. These technologies offer the potential to make crop residues more nutritionally balanced and easier for livestock to digest, thereby enhancing their value as feed resources [170].

#### 5.1.8. Feed Formulation and Nutrient Optimization

Advances in feed formulation and nutrient optimization are enabling more precise and efficient use of crop residues in livestock diets. By carefully balancing the nutrients in feed rations, livestock producers can ensure that animals receive the optimal combination of energy, protein, and fiber, leading to improved productivity and health outcomes [171].

#### 5.1.9. Sustainable Livestock Management Systems

The integration of crop residues into sustainable livestock management systems is gaining traction as a means of promoting circular agriculture. These systems emphasize the use of local resources, including crop residues, to minimize the environmental footprint of livestock production and enhance the resilience of farming communities. The successful use of agricultural crop residues in meat and milk production represents a critical component of sustainable agriculture. By transforming what is often considered waste into valuable feed resources, farmers can improve livestock productivity, reduce costs, and mitigate environmental impacts. While challenges remain, particularly in terms of treatment costs and variability in residue quality, ongoing research and technological advancements offer promising solutions. As the global demand for food continues to rise, the efficient use of agricultural crop residues will play an increasingly important role in ensuring food security, environmental sustainability, and the economic viability of farming systems [172].

## 6. Conclusions and Future Recommendations

In conclusion, agricultural crop residues and agro-industrial by-products are essential components in promoting sustainable livestock productivity. Their effective utilization presents a multifaceted approach to addressing the increasing demand for livestock products while minimizing environmental impacts. By incorporating these resources into animal feed, farmers can enhance the nutritional quality of livestock diets, reduce dependence on conventional feed grains, and lower feed costs and environmental sustainability. This not only contributes to improved livestock performance but also fosters environmental sustainability by reducing waste and methane production and promoting resource circularity.

***Key takeaway***

Agro-industrial by-products (e.g., oilseed cakes, fruit peels) can replace conventional livestock feed with cost-effective, eco-friendly alternatives, reducing the pressure on arable land. Using these wastes minimizes open burning (reducing greenhouse gas emissions) and reduces dependence on water-intensive feed crops, promoting climate-smart agriculture. Enhanced digestibility and nutrient availability stimulate milk, meat, and egg production in subtropical regions when residues are properly processed (via ensiling, urea treatment, or fermentation). Crop residues are an excellent source of biomass, which can be incorporated into livestock diets to promote a waste-to-wealth approach, improving farm profitability while reducing environmental impact.

***Future research direction/Recommendations***

Future recommendations: In order to improve the protein and energy content of low-quality residues, research should be focused on cost-effective processing methods (such as microbial or enzymatic treatment), and a residue-based diet tailored to local livestock breeds in subtropical climates would provide optimal productivity for livestock. There is a need for more data to determine how large-scale residue feeding impacts soil health, methane emissions, and water efficiency over the long term. Furthermore, educational initiatives aimed at farmers are essential to raise awareness about the potential of these alternative feed sources. Training programs that provide practical guidance on sourcing, processing, and using crop residues and by-products can empower farmers to adopt more sustainable practices. As a result of addressing these gaps, crop residues can play a crucial role in sustainable livestock production in subtropical areas.

## Figures and Tables

**Table 1:** Sugar cane and co-product composition (as a percentage of dry matter) [42].

Items	CP = Crude Protein	EE = Ether Extract;	CF = Crude Fiber	NDF = Neutral-Detergent Fiber.	ADF = Acid-Detergent Fiber.	Lignin	Total Ash
Whole sugar cane	6.0	2.1	30.6	49.6	32.5	8.4	4.7
Bagasse	3.7	1.1	44.2	92.3	81.5	25.7	5.0
Sugar cane tops	5.9	1.7	33.5	65.3	40.4	4.8	8.5

**Table 2:** Types of Agriculture Biomass [57].

Agriculture Biomass	Crops
Crop residues	Wheat straw, rice straw, barley, corn stover, and other remaining byproducts
Forage crops	Alfaalfa, clover, and other legumes provide high nutritional values
By Products	Derived from food processing, such as sugarcane bagasse, fruit pomace, and oilseed meals

**Table 3:** Chemical composition of the different crop residues [58].

Types of Crop Residues	Crude Protein	Moisture Percentage	Dry Matter
Sorghum stover	6.6%		
Sugarcane baggase	3%		89.8%
Corn stover	4.05	5.35%	93.38%
Rice straw	3.15%	8%	91.25%
Wheat straw	3.36%	4.62%	
Sugarcane tops	7.85%	55–50%	41.86%

## Data Availability

All data are included in the manuscript; however, if further information regarding data availability is needed, the corresponding author will provide it on special request.

## Supplementary Materials

The following supporting information can be downloaded at: https://www.mdpi.com/article/10.3390/ani15081184/s1.
