# Supplementary material for: The Vital Roles of Agricultural Crop Residues and Agro-Industrial By-Products to Support Sustainable Livestock Productivity in Subtropical Regions
Source: Animals (Basel). 2025 Apr 21;15(8):1184. doi: 10.3390/ani15081184 (PMC12023990; doi:10.3390/ani15081184)
Supplement: Supplementary file 1 [file animals-15-01184-s001.zip › animals-3507210-supplementary.pdf]

Supplementary file

Article

The Vital Roles of Agricultural Crop Residues and Agro-Industrial By-Products to Support the Sustainable Livestock Productivity in Subtropical Region

Ali Mujtaba Shah[1,3,¥], Huiling Zhang[2, ¥], Muhammad Shahid[4, ¥], Huma Ghazal[3], Ali Raza Shah[5], Mujahid Niaz[4] ,Tehmina Naz[6],Keshav Ghimire[7] ,Naqash Goswami[3], Wei Shi[2], Dongxu Xia[2], Hongxia Zhao[2,*.]

[1]Guangdong Provincial Key Laboratory of Animal Nutrition and Regulation, College of Animal Science, South China Agricultural University, Guangzhou, 510642, China

[2]College of Veterinary Medicine, Inner Mongolia Agricultural University, Huhhot, People's Republic of China

[3]College of Animal Science and Technology, Northwest A and F university China

[4]Laboratory of Agricultural and Food Biophysics, Institute of Biophysics, College of Science, Northwest A&F University, Yangling, Shaanxi 712100, China

[5] Khairpur College of Agricultural & Management Science, Sindh Agriculture University Tandojam Sindh Pakistan

[6]Department of Microbiology and Molecular Genetics, Woman University Multan, Pakistan

[7]College of Economics and Management, Northwest A&F University, No.3 Taicheng Road, Yangling, 712100, China

[¥]These authors contributed equally to this work

* Correspondence: Author; zhaohongxia@imau.edu.cn

S. Table 1: Use of Pineapple waste in animal feed [33].

Waste	Type	Quality	Functions
Crown leaves	Fibre	Pineapple crown leaves' cellulose nano-crystals (CNC)	CNC's strong hydrophilicity makes it useful for polymeric nanocomposites and liquid media applications.
Stem	Isolated starch (PSS)	Pineapple stem starch's pasting qualities and gelatinization	In pharmaceutical applications, modified pineapple stem starch has demonstrated promise as a tablet binder and disintegrant.
Pineapple crop waste	Silage	Pineapple waste silage (PWS) enhanced dairy cow performance.	PWS as a material meal improved body weight increase, nutritional intake, and energy balance in cattle when compared to grass-based silage.
Pineapple crop waste	Silage	The energy content, digestibility, and feed intake of cattle are all improved by pineapple waste silage.	Cattle diets can be replaced with pineapple crop waste silage.

Pineapple crop waste	Silage	Analyzing the nutritional value of silage made from pineapple crop waste for sheep.	Sheep diets can be replaced with silage made from the leftovers of pineapple crops.

S. Table 2 . Nutritional values of pineapple waste [37]

Nutrient	NFPW: Non-fermented pineapple waste	FPW: fermented pineapple waste
Ash (%)	0.04	12.88
Total soluble solids (%)	10.2	27.51
Crude fibre (g/100 g-fw)	0.60	–
Crude protein (mg/100 g)	10	0.91
Moisture (%)	91.35	72.49
Reducing sugar (%)	8.2	5
Total sugar (%)	10.2	–
Non-reducing sugar (%)	8.8	1.7

S. Table 3 . Level of inclusion of Oil Palm frond (OPF) in animals' diets [52]

Species	Feeding system	Gain g/day
Sheep	30% of OPF silage plus 70% of CONC + specific minimum block fed in stall	81.82
Rabbit	50% commercial pellet and 50% OPF pellet	78.52
Dairy cattle	70% concentrate plus 30% OPF silage	28.3
Buffalo	30% of OPF silage, 25% of PKC, 25% of copra meal, 10% of SM, 5% of FM, 35% of urea, and 2% of mineral vitamin	470.0
Deer	Half fresh OPF and Half commercial pellet	98.0
